# The DNA Sensor IFIX Drives Proteome Alterations To Mobilize Nuclear and Cytoplasmic Antiviral Responses, with Its Acetylation Acting as a Localization Toggle

**DOI:** 10.1128/mSystems.00397-21

**Published:** 2021-06-22

**Authors:** Timothy R. Howard, Marni S. Crow, Todd M. Greco, Krystal K. Lum, Tuo Li, Ileana M. Cristea

**Affiliations:** a Department of Molecular Biology, Princeton University, Princeton, New Jersey, USA; NIAID, NIH

**Keywords:** DNA sensing, HSV-1, IFIX, innate immunity, lysine acetylation, mass spectrometry, posttranslational modification, proteomics

## Abstract

DNA sensors are critical components of innate immunity that enable cells to recognize infection by pathogens with DNA genomes. The interferon-inducible protein X (IFIX), a member of the PYHIN protein family, is a DNA sensor capable of promoting immune signaling after binding to double-stranded DNA (dsDNA) within either the nucleus or cytoplasm. Here, we investigate the impact of IFIX on the cellular proteome upon introduction of foreign DNA to the nucleus or the cytoplasm as well as regulatory hubs that control IFIX subcellular localization. Using quantitative mass spectrometry, we define the effect of CRISPR-mediated IFIX knockout on nuclear and cytoplasmic proteomes in fibroblasts. Proteomes are probed in response to either nuclear viral DNA, during herpes simplex virus 1 (HSV-1) infection, or cytoplasmic viral DNA, following transfection with dsDNA derived from vaccinia virus (VACV 70-mer). We show that IFIX broadly impacts nuclear and cytoplasmic proteomes, inducing alterations in the abundances of immune signaling, DNA damage response, and vesicle-mediated transport proteins. To characterize IFIX properties that regulate its localization during DNA sensing, we perform deletion and mutagenesis assays. We find that IFIX contains a multipartite nuclear localization signal (NLS) and highlight the main contributing motif for its nuclear localization. Using immunoaffinity purification, we identify IFIX acetylation and phosphorylation sites. Mutations to acetyl or charge mimics demonstrate that K138 acetylation, positioned within the NLS, affects nuclear localization. Altogether, our study establishes a mechanism regulating IFIX subcellular localization and contextualizes this localization with the involvement of IFIX in host cell responses to pathogenic DNA.

**IMPORTANCE** Mammalian cells must be able to detect and respond to invading pathogens to prevent the spread of infection. DNA sensors, such as IFIX, are proteins that bind to pathogen-derived double-stranded DNA and induce antiviral cytokine expression. Here, we characterize the host proteome changes that require IFIX during both viral infection and DNA transfection. We show IFIX mobilizes numerous pathways and proteome alterations within the nucleus and the cytoplasm, pointing to a multifunctional protein with roles in immune signaling, DNA damage response, and transcriptional regulation. We next interrogate the IFIX domains required for nuclear localization, discovering its regulation via a multipartite nuclear localization motif. The acetylation of this motif promotes IFIX cytoplasmic localization, in agreement with its detection of pathogenic DNA in both the nucleus and the cytoplasm. This study established NLS acetylation as a conserved mechanism for regulating the localization of nuclear DNA sensors from the PYHIN family of proteins.

## INTRODUCTION

The detection of pathogens by host cells is a critical first step for the mammalian immune system in warding off infections. The ability of cells to recognize and respond to invading microbes relies on the use of an array of pattern recognition receptors (PRRs) that are capable of detecting a multitude of pathogen-associated molecular patterns (PAMPs), including proteins, lipids, sugars, and nucleic acids (1). Double-stranded DNA (dsDNA), originating from bacteria, DNA viruses, and some RNA viruses (i.e., retroviruses), is a common PAMP encountered by cells (2). To detect foreign dsDNA, cells employ a class of PRRs known as DNA sensors that can recognize pathogen-derived dsDNA and activate immune programs to stymie the spread of infections. The localization of pathogen DNA within the host cell varies depending on the type of infection and the replication cycle of the DNA virus (2, 3). For example, the dsDNA genome of a herpesvirus such as herpes simplex virus 1 (HSV-1) is deposited and replicated in the nucleus (4), while that of a poxvirus like vaccinia virus (VACV) remains in the cytoplasm (5). Thus, to ensure successful identification of an infection, DNA sensors are localized in different subcellular locations, including in the cytosol, within endosomes, and in the nucleus (2).

PYHIN proteins comprise a family of proteins that has gained attention in recent years for their ability to detect pathogenic DNA both within the cytoplasm and nucleus (3). PYHIN proteins are named as such because of their characteristic N-terminal pyrin domains (PY), which mediate homotypic protein interactions (6, 7), and the one or more C-terminal HIN200 (hematopoietic expression, interferon-inducible nature, and nuclear localization) domains, which bind to dsDNA in a sequence-independent manner (8). Listed in order of their discovery as DNA sensors, PYHIN family members that are expressed in human cells include absent in melanoma 2 (AIM2) (9), interferon-inducible protein 16 (IFI16) (10), and interferon-inducible protein X (IFIX) (11). A number of studies have focused on characterizing how AIM2 recognizes cytoplasmic DNA, initiates the formation of a multisubunit immune signaling complex known as the inflammasome, and launches a form of programmed cell death termed pyroptosis (7–9, 12, 13). Additionally, in recent years, IFI16 has emerged as a DNA sensor in the nucleus, capable of relocalizing to the nuclear periphery to bind to incoming dsDNA genomes of the herpesviruses HSV-1 and human cytomegalovirus (HCMV). Upon binding to viral DNA, IFI16 was shown to exert its antiviral functions via both repressing the expression of viral genes and inducing antiviral cytokine expression (10, 14–20). Furthermore, IFI16 has also been found to sense viruses that utilize DNA intermediates in their replication cycles, such as human immunodeficiency virus 1 (HIV-1) (21, 22). In contrast to AIM2 and IFI16, the most recently discovered DNA sensor in the PYHIN family, IFIX (11), also known as PYHIN1, remains relatively uncharacterized.

IFIX was initially identified as a tumor suppressor, displaying pRB- and p53-independent antiproliferative properties as well as reduced expression levels in most human breast tumors and breast cancer cell lines (23). More than a decade after this discovery, our laboratory characterized IFIX as a nuclear DNA sensor during HSV-1 infection (11). In agreement with its DNA sensing abilities, *in vitro* assays showed that IFIX binds dsDNA in a sequence-independent manner, while during infection with HSV-1 it associates with different regions of the viral genome (11). Further investigations pointed to similarities to IFI16, as IFIX displayed changes in its intranuclear distribution early in HSV-1 infection, becoming recruited to nuclear periphery puncta that colocalized with incoming viral genomes (24). Its antiviral functions were reflected in the impact of IFIX levels on HSV-1 production, as IFIX overexpression in an inducible HEK293 cell system and IFIX knockdown in fibroblasts resulted in decreased and increased virus titers, respectively. Characterization of IFIX protein interactions revealed its association with transcriptional regulators during HSV-1 infection, including members of five friends of methylated chromatin target of Prmt (5FMC), which, via further assays, were implicated in the IFIX regulation of HSV-1 gene expression (24). More recently, the antiviral role of nuclear IFIX was expanded with the discovery of its ability to also restrict human immunodeficiency virus (HIV) replication as well as the production of the polyomavirus simian virus 40 (SV40), an oncogenic DNA virus in human macrophages and CD4^+^ T cells by sequestering the host transcription factor Sp1 (25). Its inhibition of HIV-1 replication was demonstrated in human macrophages and CD4^+^ T cells and linked to the ability of the IFIX to sequester the host transcription factor Sp1. The IFIX-Sp1 interaction was mediated by the Sp1 Zn-finger domain and the IFIX region containing the pyrin domain and the putative nuclear localization signal (NLS) motifs (25).

Interestingly, although IFIX exhibits a pronounced nuclear localization, its ability to surveil for pathogenic DNA was shown to extend to the cytoplasm (11). Transfection with a 70-bp vaccinia virus DNA fragment (VACV70) in HEK293 cells that express IFIX resulted in elevated beta inteferon (IFN-β) expression compared to cells lacking IFIX. Colocalization of IFIX and VACV70 was also observed in the cytoplasm of HEK293 cells, implicating this predominantly nuclear protein in cytoplasmic DNA sensing (11). However, the mechanisms that regulate the movement of IFIX between the nucleus and the cytoplasm remain unknown. Furthermore, although IFIX was reported to contribute to transcriptional regulation and immune signaling, its broad impact on the cellular proteome during infection has not yet been investigated.

Here, we investigate the regulation of IFIX subcellular localization as well as its impact on the cellular proteome upon introduction of nuclear or cytoplasmic foreign DNA. Using CRISPR-mediated knockouts and quantitative mass spectrometry, we define the effect of IFIX knockout on nuclear and cytoplasmic proteome compositions. We assess these proteome alterations in response to host cell exposure to both nuclear viral DNA, during early stages of HSV-1 infection, and cytoplasmic viral DNA following transfection with VACV70. We find that IFIX knockout has a broad impact on nuclear and cytoplasmic proteomes, inducing alterations in the abundances of immune signaling factors, DNA damage response proteins, and vesicle-mediated transport proteins. We also observe slight compensatory and coregulatory effects on the levels of the other two nuclear DNA sensors, IFI16 and hnRNPA2B1 (heterogeneous nuclear ribonucleoprotein A2/B1) (26) as well as in the levels of OASL (2′-5′-Oligoadenylate Synthetase Like), a protein linked to RNA sensing pathways (27). Given this expansive impact of IFIX on both nuclear and cytoplasmic proteomes as well as its ability to sense foreign DNA in both compartments, we go on to interrogate the mechanisms contributing to its subcellular localization. Similar to IFI16, we find that IFIX contains a multipartite NLS. Using mutagenesis, we identify the predominant motif (motif-2) that drives its nuclear localization. Additionally, we map posttranslational modifications via acetylation and phosphorylation present on IFIX. Using site-directed mutagenesis to either acetyl or charge mimics, we show that IFIX K138 acetylation, positioned within the NLS motif-2, impedes nuclear localization. This finding expands our previous observation for functional NLS acetylation for IFI16, demonstrating that lysine acetylation within the NLS represents a conserved mechanism for regulating the localization of nuclear DNA sensors from the PYHIN family of proteins.

## RESULTS

### IFIX displays dynamic localization within the nucleus during immune signaling.

**FIG 1:**
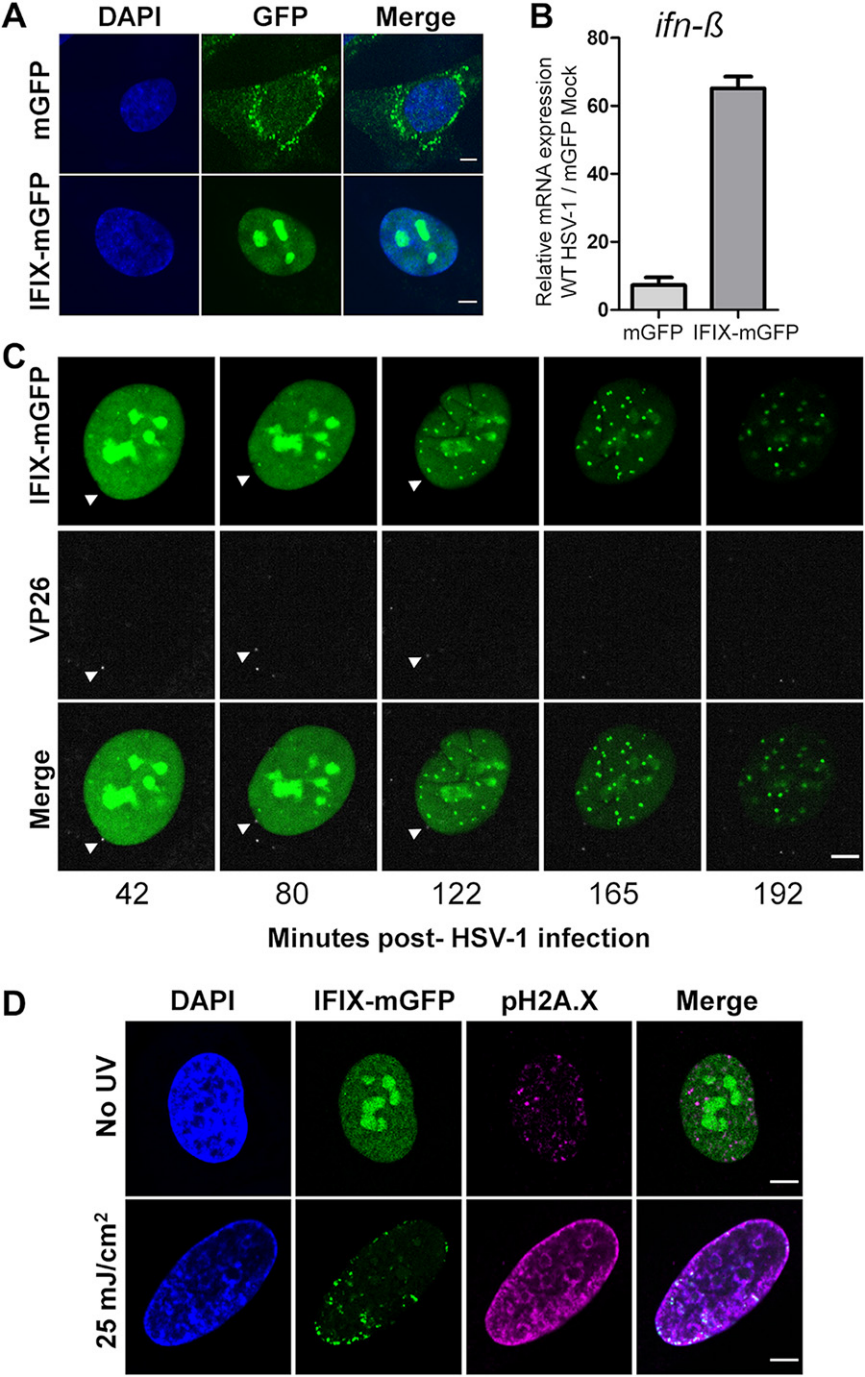
Nuclear localization dynamics of IFIX upon stimulation by HSV-1 dsDNA or damaged DNA. (A) Stable HFF cell lines were generated with free mGFP (top) and mGFP-tagged IFIX (bottom). (B) *Ifn-β* mRNA levels in mGFP and IFIX-mGFP cells infected with WT HSV-1 (MOI of 10) at 6 hpi. Data were normalized to *gapdh* and then replicate normalized before computing fold difference over the mGFP mock condition. Values are means ± standard deviations (SD) (*n *= 2). (C) IFIX-mGFP HFFs were infected with HSV-1::*mrfp-vp26* (MOI of 10). mRFP signal is pseudocolored gray. White arrow indicates viral capsid docking on nucleus. (D) IFIX-mGFP HFFs were left untreated or treated with 25 mJ/cm^2^ UV and then fixed and stained 24 h later. Phosphorylated H2A.X indicating dsDNA breaks is shown in the magenta channel. Scale bars, 5 μm in all panels.

To facilitate investigations into the DNA sensing role of IFIX during HSV-1 infection, we utilized primary human fibroblasts (HFFs) stably expressing either monomeric green fluorescent protein (mGFP) or mGFP-tagged IFIX (IFIX-mGFP), as in reference 24. As expected, IFIX-mGFP localized predominantly to the nucleus and exhibited nucleolar enrichment (Fig. 1A). Our previous studies revealed that this overexpression of IFIX-mGFP reduces the expression of HSV-1 genes during infection compared to control mGFP cells (24). The related nuclear PYHIN protein IFI16 has also been shown to inhibit viral gene expression while also inducing the expression of antiviral cytokines during HSV-1 infection (19). Therefore, to determine whether IFIX can similarly promote interferon expression in response to HSV-1 infection, we measured the production of IFN-β mRNA at 6 h postinfection (hpi) during wild-type (WT) HSV-1 infection. Levels of IFN-β mRNA were ∼9-fold higher in IFIX-mGFP than mGFP fibroblasts (Fig. 1B), indicating that IFIX can indeed aid cytokine induction during HSV-1 infection.

We have previously observed IFIX redistribution within the nucleus upon HSV-1 infection (24). To gain a better temporal resolution of this redistribution and to monitor its dependence on the deposition of viral DNA into the nucleus, we used live-cell microscopy to investigate the dynamics of IFIX localization upon infection of IFIX-mGFP fibroblasts with HSV-1::*mrfp-vp26*. This virus strain has the capsid protein VP26 tagged with monomeric red fluorescent protein (mRFP), thereby allowing us to monitor the movement of the virus capsid in infected cells. During the early stages of infection, the HSV-1 capsid docks at the nuclear pore complex and injects the viral genome into the nucleus as naked dsDNA, which afterwards leads to the expression of viral immediate-early genes (4). We observe the docking of the virus capsid to the nuclear periphery as early as ∼40 min postinfection (Fig. 1C, left). In response to HSV-1 genome deposition, IFIX became relocalized within the nucleus. The nucleolar IFIX signal was substantially reduced in conjunction with an increased number of nuclear IFIX-containing puncta as the infection progressed (Fig. 1C). Therefore, the docking of the viral capsid at the nuclear periphery can act as a signal that induces changes in IFIX localization, possibly activating the IFIX nucleolar reservoirs.

To gain further insights into the types of signals that can promote this IFIX redistribution, we asked whether the incoming viral DNA activates host DNA damage response pathways. This hypothesis was also driven by the known role of IFIX as a tumor suppressor (23) and our previous finding that IFIX associates with several proteins involved in the DNA damage response (DDR) and DNA repair pathways, including the DNA-PK subunits XRCC5 and XRCC6 (11). Therefore, we monitored the IFIX-mGFP localization in fibroblasts following induction of cellular DNA damage using UV irradiation. We found that IFIX forms nuclear aggregates that colocalize with sites of double-stranded DNA breaks (marked by phosphorylated H2AX) 24 h after UV treatment (Fig. 1D). The puncta formed in response to DNA damage appeared more jagged and less regular than those formed during HSV-1 infection and were concentrated around the nuclear periphery. This localization agrees with the knowledge that DNA double-strand breaks relocalize to the nuclear periphery prior to their repair (28). Therefore, our findings point to the ability of IFIX to be recruited to sites of damaged DNA, suggesting a function in resolution of DNA damage.

### IFIX drives both nuclear and cytoplasmic proteome changes early in HSV-1 infection.

**FIG 2:**
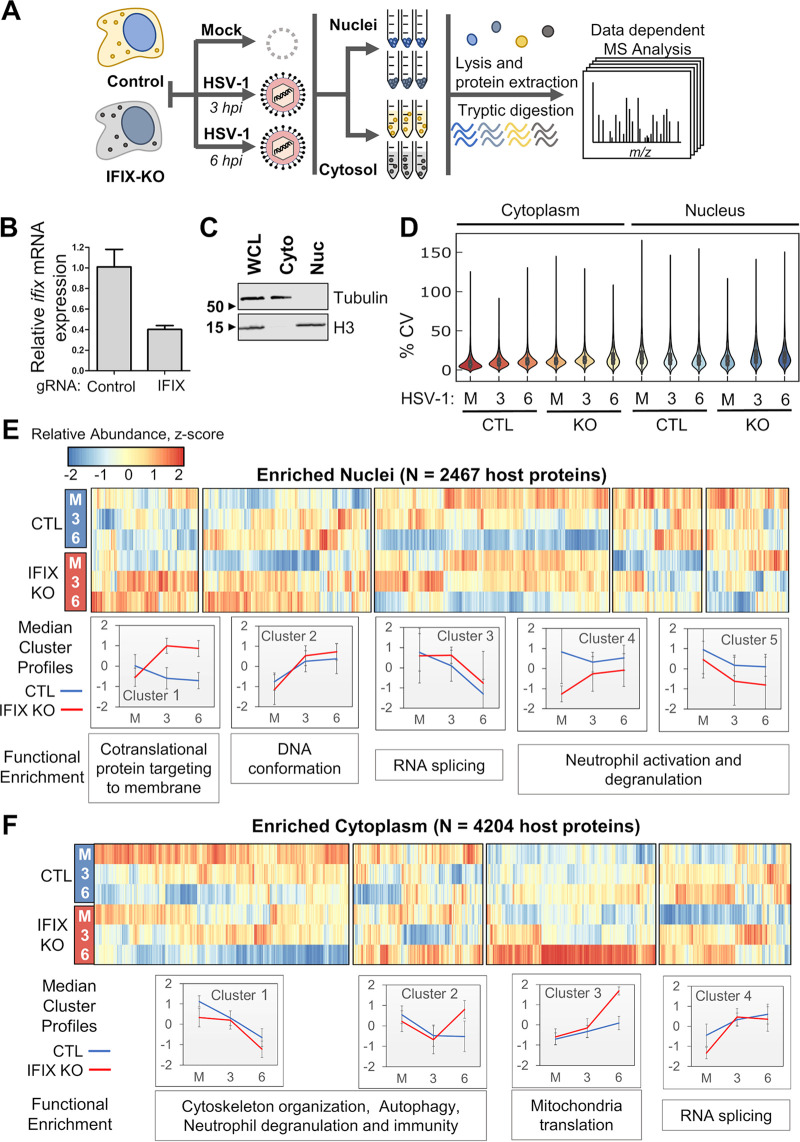
Subcellular proteomic analysis of IFIX-mediated response to HSV-1 infection. (A) Workflow for analysis of nuclear and cytoplasmic proteomes in control and IFIX-KO HFFs. (B) The efficiency of IFIX knockout was determined by measuring *ifix* mRNA levels via RT-qPCR. mRNA levels were normalized to *β-actin*. Values are means ± SD from three technical replicates. (C) Western blot demonstrating the efficiency of nuclear-cytoplasmic fraction in control HFFs. (D) Quantitative MS protein abundance coefficient of variance for cytoplasm and nucleus samples from mock (M), 3 hpi (3), and 6 hpi (6) in IFIX KO and control cells. (E and F) Hierarchical clustering from nuclear (E) and cytoplasmic (F) samples of the average MS protein abundances (*n *= 3 replicates), which were row centered and scaled. Cluster profiles were calculated based on the median centered/scaled abundances with median absolute deviation error bars.

To gain a better understanding of the impact of IFIX on nuclear organization and processes, we utilized a mass spectrometry (MS)-based approach to examine IFIX-dependent alterations in the nuclear and cytoplasmic proteomes during the early stages of HSV-1 infection (Fig. 2A). We first constructed control and IFIX knockout human fibroblast cell lines (referred to as control and IFIX-KO HFF, respectively) using CRISPR/Cas9-mediated knockouts. IFIX knockout efficiency was determined by measuring *ifix* mRNA expression via quantitative PCR (qPCR) (Fig. 2B). Cells were then mock infected (i.e., uninfected) or infected with WT HSV-1 (multiplicity of infection [MOI] of 5) and collected at 3 and 6 hpi. To capture localization-dependent proteome changes, we performed nuclear-cytoplasmic fractionation before the MS analysis. The efficiency of fractionation was confirmed by Western blotting (Fig. 2C).

Nanoliquid chromatography paired with quantitative label-free mass spectrometry (nLC-MS) was used to investigate changes in protein abundance between control and IFIX-KO cells during HSV-1 infection. Overall, 2,467 and 4,204 proteins were reproducibly quantified from nuclear and cytoplasm-enriched fractions, respectively (coefficients of variation shown in Fig. 2D). While we observed the expected enrichments for nuclear and cytoplasmic proteins, we also noted the presence of some membrane-bound and mitochondrial proteins within the nuclear fraction, a caveat common for this type of biochemical fractionation. Average protein abundances for mock, 3, and 6 hpi in IFIX-KO and control HFFs were visualized in heatmaps using clustering by relative abundance trends, which were associated with specific enriched functions (Fig. 2E and F; see also Fig. S1A and C in the supplemental material). Within the nucleus, we observed that protein cluster 1 was associated with cotranslational protein targeting to membranes and reflected median abundance changes that were highly upregulated during infection in IFIX-KO cells (Fig. 2E, cluster 1), while in cells expressing IFIX, protein abundances were slightly lower at both 3 and 6 hpi. In contrast, cluster 2 was enriched for proteins associated with regulation of DNA conformation (Fig. S1A and B). We also identified a cluster of proteins within the cytoplasm with a similar trend that was enriched for the GO term mitochondrial translation (Fig. 2F, cluster 3). Compared to these two clusters, the remaining clusters showed distinct abundance trends but were more similar between IFIX-KO and IFIX-expressing cells. Proteins associated with RNA splicing were quantified in both nuclear (Fig. 2E, cluster 3) and cytoplasmic fractions (Fig. 2F, cluster 4). Interestingly, the median protein abundance trends for the RNA splicing-associated clusters showed infection-dependent increases in nuclei and opposing decreases in the cytoplasm. In addition, given our previous finding that IFIX restricts HSV-1 replication and promotes immune signaling (11, 24), we expected that the abundance of proteins involved in immune response would be decreased in IFIX-KO HFFs compared to control cells. Our data pointed to a cluster of proteins involved in immunity that had decreased abundances upon IFIX-KO, a cluster that also contained proteins related to autophagy and neutrophil degranulation (Fig. 2F, cluster 1). As HSV-1 is known to possess mechanisms that suppress host immune signaling, it is unsurprising that a decrease in immunity proteins was also observed in control cells, and this phenotype was slightly exacerbated in IFIX-KO cells.

10.1128/mSystems.00397-21.2 FIG S1 Gene ontology and network analysis of nuclear and cytoplasm IFIX-dependent protein profiles in HSV-1 infected cells. (A) Proteins assigned to clusters 1 – 5 from the nuclear proteome (see Fig. 2E) were analyzed by comparative overrepresentation analysis of GO Biological Processes using clusterProfiler and visualized by the dot plot function. The top three GO terms for each cluster are shown, with the total number of annotated proteins across all clusters in parentheses. The dot size and color reflect the proportion of annotated proteins in each cluster and overrepresentation significance, respectively. (B) STRING functional network of annotated proteins in the DNA conformational change GO BP that were identified in (A). Node color and edge thickness represent the protein’s assigned cluster and STRING combined score magnitude (0.4 – 1). (C) Same as (A), except comparative overrepresentation was performed with clusters 1 – 4 of the cytoplasmic proteome (see Fig. 2F). Download FIG S1, TIF file, 1.6 MB. Copyright © 2021 Howard et al. 2021 Howard et al. https://creativecommons.org/licenses/by/4.0/ This content is distributed under the terms of the Creative Commons Attribution 4.0 International license.

**FIG 3:**
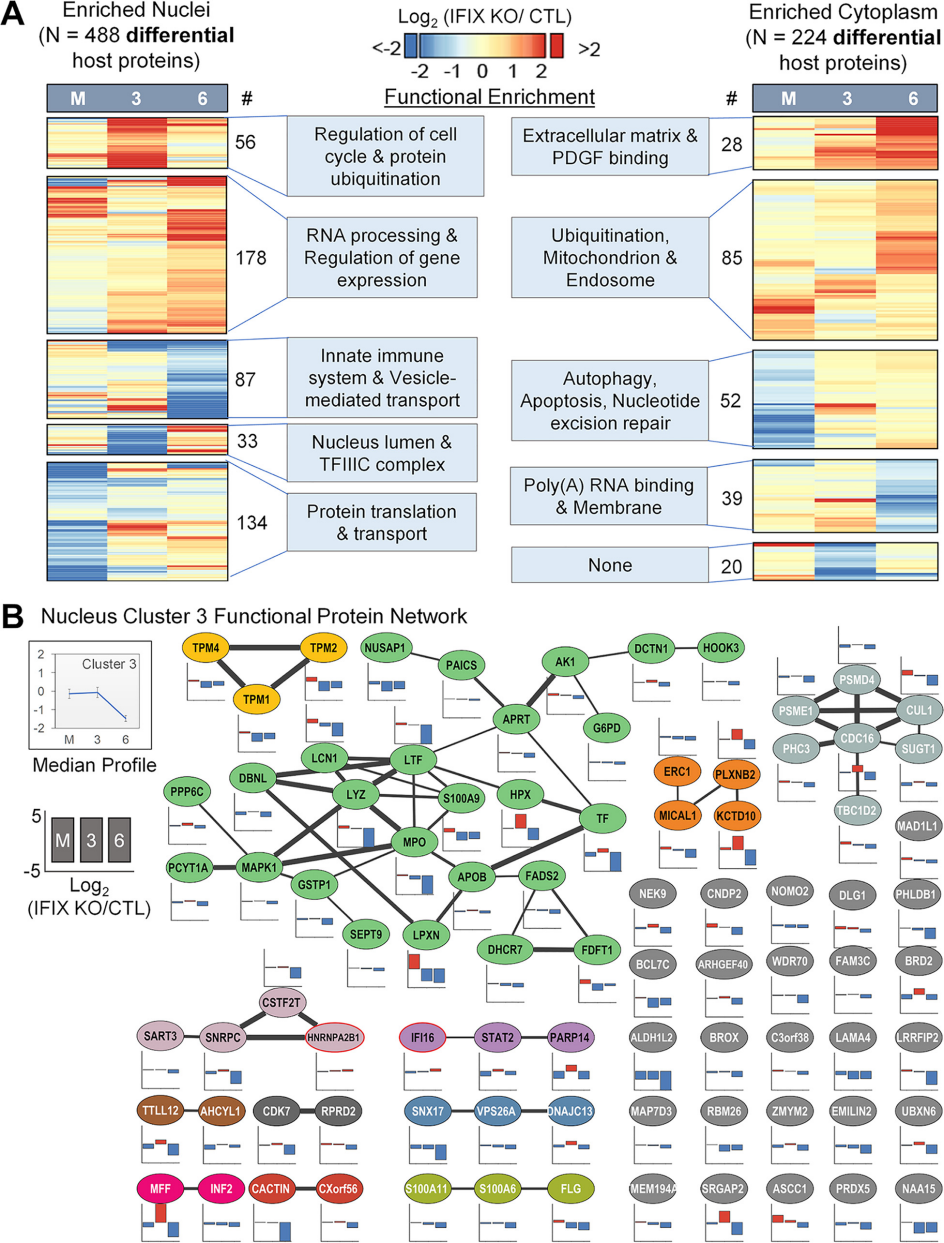
IFIX-dependent modulation of distinct protein networks by differential proteome analysis. (A) Hierarchical clustering of log_2_ (IFIX-KO/CTL) from mock (M), 3-hpi (3), and 6-hpi (6) conditions in the nucleus (left) and cytoplasm (right). (B) STRING functional protein network analysis performed on the 87 proteins from nuclear cluster 3 in panel A. Node color and edge thickness represent distinct neighborhood clusters and STRING combined score (0.4 to 1). For each node, an up-down bar graph shows log_2_ (IFIX-KO/CTL) values.

To identify the subset of high-confidence proteins with differential abundance after IFIX knockout, we assessed the magnitude and reproducibility of the abundance changes. This was achieved by determining pairwise fold change and *P* value thresholds separately for mock, 3-, and 6-hpi treatment comparisons of IFIX-KO and control cells, which maintain a specific target false discovery rate (FDR) (2.5%; see Materials and Methods). Across all conditions, we identified 488 and 224 differential proteins in enriched nuclei and cytoplasm, respectively (Table S1). Clustering differential proteins by their fold change values and subsequent GO enrichment showed GO terms similar to those in Fig. 2 but with greater sensitivity of detection of overrepresented terms (Fig. 3A). For example, in the nuclear proteome, 56 proteins with increased abundance in IFIX KO versus control at 3 hpi (cluster 1) were enriched in the regulation of the cell cycle and protein ubiquitination. Notably, we identified a cluster of proteins decreased at 6 hpi in IFIX-KO cells that were associated with the innate immune system and vesicle-mediated transport (Fig. 3A, left heatmap, cluster 3). For example, levels of CACTIN, LRRFIP2 (Leucine-rich repeat flightless-interacting protein 2), TTLL12 (Tubulin–tyrosine ligase-like protein 12), ERC1 (ELKS/Rab6-interacting/CAST family member 1), and STAT2 are decreased upon IFIX KO at 6 hpi compared to 3 hpi (Fig. 3B). Overall, this suggests that IFIX helps to induce immune regulatory proteins during viral infection. While the other two known nuclear DNA sensors, IFI16 and hnRNPA2B1, were not part of this cluster, we interrogated their network connectivity and abundance changes (Fig. 3B, red node border). IFI16 and hnRNPA2B1 have similar abundance profiles during HSV-1 infection, undergoing modest increases upon IFIX-KO at 6 hpi (1.6- and 1.4-fold, respectively).

10.1128/mSystems.00397-21.1 TABLE S1 MS abundances and differential analysis for proteins identified in the nuclear and cytoplasmic fractions of cells mock infected, HSV-1 infected for 3 hpi, HSV-1 infected for 6 hpi, and VACV70 transfected. Download Table S1, XLSX file, 5.6 MB. Copyright © 2021 Howard et al. 2021 Howard et al. https://creativecommons.org/licenses/by/4.0/ This content is distributed under the terms of the Creative Commons Attribution 4.0 International license.

In support of our observations of a possible involvement for IFIX in DNA damage repair, IFIX knockout in mock-infected cells resulted in the downregulation of proteins connected to autophagy, apoptosis, and nucleotide excision repair (Fig. 3A, right, cluster 3). These proteins included DNA damage-binding protein 2 (DDB2), DNA mismatch repair protein Mlh1, and TFIIH basal transcription factor complex helicase XPD subunit (ERCC2) as well as the apoptotic regulators XAF1, PLEKHF1, and SAP30BP. Overall, these quantitative proteomics measurements reveal roles for IFIX in basal protein homeostasis as well as its ability to broadly contribute to alterations in nuclear and cytoplasmic proteomes and immune modulation during HSV-1 infection.

### IFIX localizes to cytoplasmic VACV dsDNA and contributes to the rewiring of the cytoplasmic proteome in response to foreign DNA.

**FIG 4:**
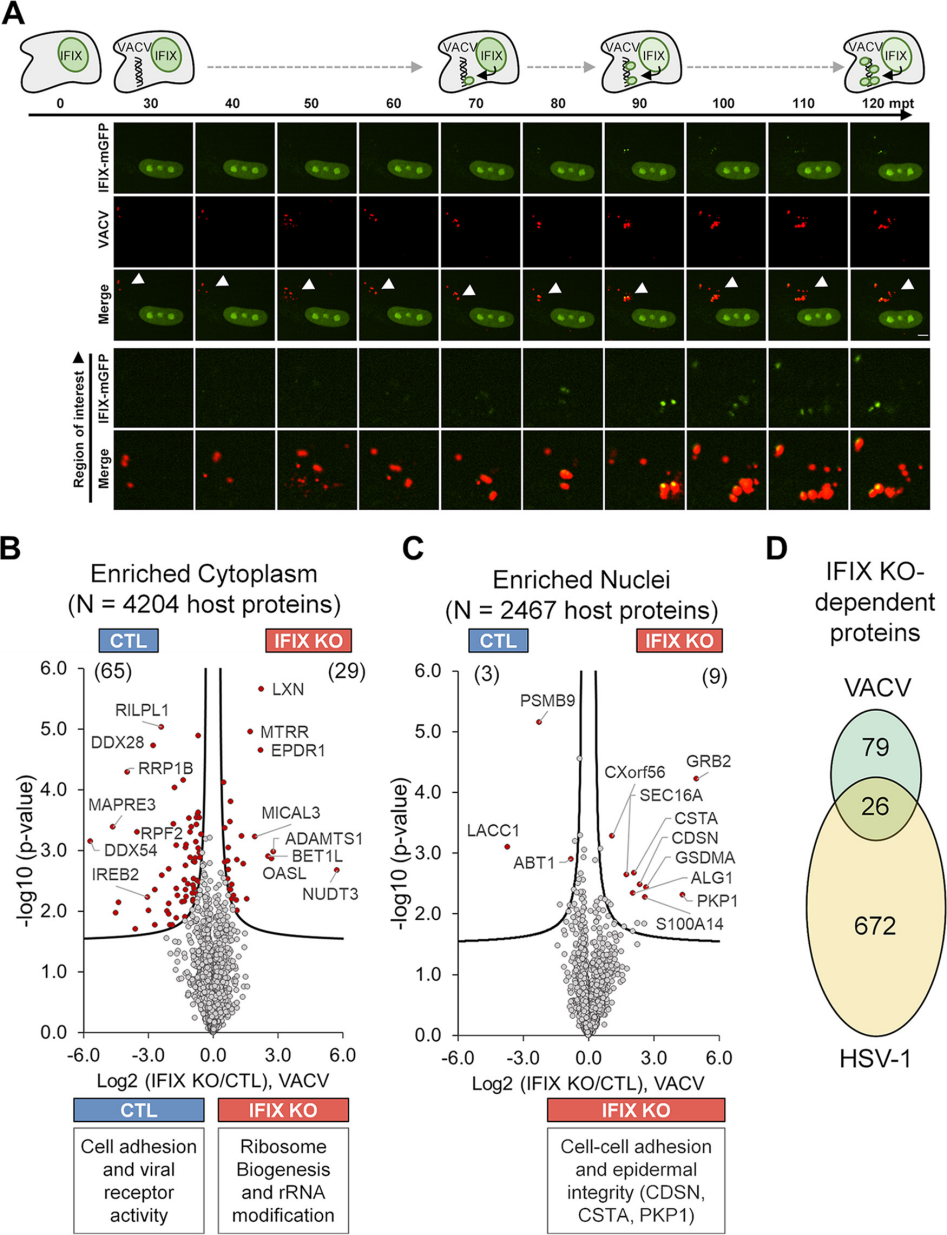
Subcellular proteomic analysis of IFIX-mediated response to transfection with VACV dsDNA. (A) Cy5-labeled VACV was transfected into IFIX-mGFP HFFs and visualized by live-cell confocal microscopy from 30 min through 2 h posttransfection (mpt). Yellow signal in merged images show colocalization of IFIX-mGFP and Cy5-VACV. Arrows indicate 12-μm by 12-μm regions of interest shown below. Scale bars, 5 μm. (B and C) Differential proteome analysis of VACV-treated IFIX-KO and control cytoplasmic (B) and nuclear (C) proteomes visualized by Volcano plots. Curves reflect *P* values and log_2_ fold change thresholds at 2.5% FDR. Significant and nonsignificant proteins are indicated by red and gray data points, respectively. The numbers of significant up (IFI-KO)- and down (CTL)-regulated proteins are indicated in parentheses. Representative functions associated with the differential proteins are shown below the plots. (D) The number of unique and common differential proteins (up- and downregulated) detected during HSV-1 infection (Fig. 3) and VACV treatment.

Given our finding that IFIX broadly impacts the cellular proteome during HSV-1 infection, in conjunction with our previous discovery that IFIX can also translocate to the cytosol to sense transfected DNA in the cytoplasm (11, 24), we next further investigated the role and impact of IFIX within the cytoplasm. Our prior observation of cytoplasmic DNA sensing was made in 293 cells inducible for IFIX-GFP expression, so we sought to investigate its cytoplasmic function within a more biologically relevant system by using IFIX-mGFP fibroblasts. We constructed Cy5-labeled VACV 70-mer that we transfected into fibroblasts as a source for cytoplasmic viral dsDNA. IFIX displayed colocalization with VACV70 within cytoplasmic aggregates by approximately 1 h posttransfection (Fig. 4A). Having established that IFIX senses VACV dsDNA within the cytoplasm, we next investigated its impact on proteome changes upon this cytoplasmic immune stimulation. Using a workflow similar to that in Fig. 2A, we transfected control and IFIX-KO fibroblasts with VACV70, collected samples after 6 h, and performed nuclear-cytoplasmic fractionation before MS analysis. Our results showed that, within the cytoplasm of IFIX-KO cells, 65 proteins were significantly downregulated while 29 proteins were upregulated (Fig. 4B). Downregulated proteins were involved in cell adhesion and viral receptor activity, and upregulated proteins were associated with ribosome biogenesis and rRNA modification. Given the enrichment of IFIX in nucleoli, this observation supports the hypothesis that IFIX is involved in homeostasis of ribosome biogenesis. Additionally, our laboratory previously characterized the negative regulation of the prominent cytoplasmic DNA sensor cGAS by OASL, a member of the oligoadenylate synthase family of proteins linked to double-stranded RNA sensing in the cytoplasm (27, 29). The increased OASL abundance upon IFIX knockout indicates the possible presence of a cross talk between these two proteins. In agreement with the presence of cytoplasmic DNA stimuli, the number of differentially expressed proteins in the nucleus was much lower than that in the cytoplasm: 3 proteins were downregulated in IFIX-KO cells and 9 were upregulated, and these proteins were involved in cell-cell adhesion and epidermal integrity (Fig. 4C).

Comparison of the proteome changes driven by HSV-1 infection and VACV70 transfection indicated that most of the alterations were not shared between these two perturbations. However, we also observed a subset of proteins that displayed significant IFIX-dependent expression changes under both conditions. Of the 105 differentially expressed proteins in VACV70 transfection, 26 were shared with the set of 698 IFIX-dependent proteins in HSV-1 infection (Fig. 4D), including OASL and DDX60, the latter of which has a role in type I IFN expression during DNA virus infection (30). These results suggest that IFIX functions in a localization-dependent manner, contributing to unique proteome alterations within the nucleus and cytoplasm in response to foreign DNA.

### Subcellular localization of IFIX is regulated by a multipartite nuclear localization signal.

**FIG 5:**
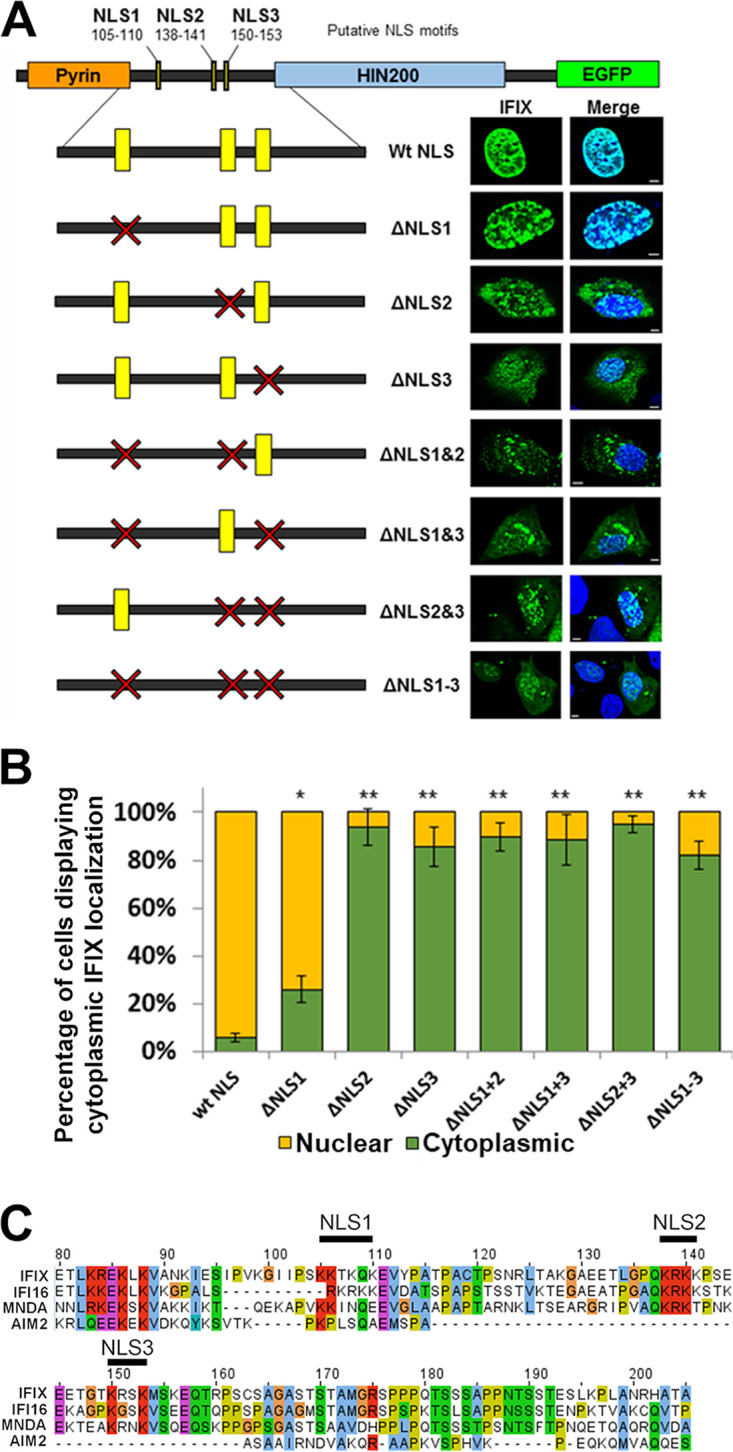
IFIX NLS motifs 2 and 3 contribute to its nuclear localization. (A, left) Schematic of putative IFIX NLS locations within the protein and the NLS deletion constructs that were generated. Yellow bars represent NLS locations; red X indicates the NLS was deleted. (Right) Representative confocal microscopy images of U2OS cells that were transfected with either wild-type or NLS deletion IFIX. Scale bars, 5 μm. (B) Quantification of IFIX subcellular localization (percentage of cells). Error bars represent SD from 3 biological replicates; *n *≥ 50 cells; *, *P* ≤ 0.05; **, *P* ≤ 0.005; in relation to the wild type. (C) Amino acid alignment of the IFIX linker region with the other human PYHIN proteins. Putative IFIX NLS motifs are indicated above. Alignment performed with Clustal Omega and visualized in Jalview with ClustalX color scheme.

Given that a small subset of IFIX translocates from the nucleus to sense VACV70 within the cytoplasm and induce interferon response (Fig. 4B) (11), we reasoned that IFIX subcellular localization must be tightly regulated. The ability for localization-dependent sensing has also been reported for the other PYHIN sensor, IFI16 (10), and we previously characterized a bipartite IFI16 NLS located in the linker region between the PY and HIN200 domains (31). Thus, we bioinformatically searched for putative NLS signature motifs within the amino acid sequence of IFIX by using the software NLSmapper (32). The scores given by NLSmapper reflect subcellular localization, where a higher number indicates increased likelihood that the protein is associated with the nucleus (e.g., scores of ≥8 are considered exclusively nuclear and scores of 3 to 4 indicate possible dual localization to the nucleus and cytoplasm). Both a monopartite NLS (135-GPQKRKKPSE-144, score 9) and an extended bipartite NLS (135-GPQKRKKPSEETGTKRSKMS-154, score 11.9) were predicted (Fig. S2B and C). Other bipartite regions with lower scores were also predicted, albeit with much lower scores, such as the 80-ETLKREKLKVANKIESIPVKGIIPSKKTKQK-110 region, with a score 3.4 (Fig. S2C). As nuclear import is dependent on karyopherins, which recognize basic residues within an NLS (33, 34), we focused on the predicted NLS motifs that are rich in lysine and arginine residues. Specifically, we tested the IFIX regions 105-KKTKQK-110, 138-KRKK-141, and 150-KRSK-153, which we labeled NLS motif-1, -2, and -3, respectively (Fig. 5A).

10.1128/mSystems.00397-21.3 FIG S2 Bioinformatic analysis of putative IFIX NLS motifs and analysis of IFIX NLS mutant expression levels. (A) NLSmapper predicted NLSs in IFIX FASTA query sequence. Red highlights areas in IFIX protein amino acid sequence where NLSs may exist. (B) NLS mapper predicted monopartite NLSs with their associated scores. The score indicates the likelihood of nuclear localization; the higher the number, the higher the probability the protein is nuclear. (C) Same as panel B but represents bipartite motifs. Panels A to C are from NLSmapper: http://nls-mapper.iab.keio.ac.jp/cgi-bin/NLS_Mapper_form.cgi. (D) Average fluorescence intensity of IFIX-GFP was quantified from images of WT and ΔNLS mutant IFIX-EGFP constructs transiently transfected in U2OS cells. Regions of interest (ROIs) were drawn around the nucleus when cytoplasmic IFIX-GFP was absent and around the entire cell when cytoplasmic IFIX-GFP was present. Mean fluorescence intensities within the ROIs were measured, normalized by the ROI area, and then scaled between 0 and 1 among all samples. Bars represent 5 to 95 percentile; *n *≥ 12 cells. Download FIG S2, TIF file, 2.7 MB. Copyright © 2021 Howard et al. 2021 Howard et al. https://creativecommons.org/licenses/by/4.0/ This content is distributed under the terms of the Creative Commons Attribution 4.0 International license.

To test whether one or several of these NLS motifs contribute to the IFIX nuclear localization, we generated GFP-tagged IFIX NLS deletion mutants. Constructs were made for all the eight possible NLS deletion combinations of the three motifs and denoted by ΔNLS and the motif number(s). These constructs were then transfected into U2OS cells, and the IFIX localization was assessed by direct fluorescence confocal microscopy. Quantification of IFIX fluorescent signal showed similar levels of protein expression for WT, ΔNLS1, and ΔNLS2 and a slight increase in signal for ΔNLS3 (Fig. S2D). Although no construct caused IFIX to be exclusively cytoplasmic, all deletion constructs led the presence of some degree of cytoplasmic IFIX (Fig. 5A). In quantifying the effect of these deletions, ΔNLS1 impacted IFIX localization the least, with most cells (74%) still displaying nuclear IFIX (Fig. 5B). However, ΔNLS2 and ΔNLS3 alone or in combination exhibited 80% or more transfected cells having a subset of cytoplasmic IFIX (Fig. 5B). The simultaneous double deletion of NLS2 and NLS3 did not show a further decrease in nuclear IFIX, as would be expected if these motifs functioned independently from each other to support IFIX localization. This suggests that the IFIX nuclear localization is regulated by a multipartite NLS. Another NLS region was predicted by NLSmapper within the IFIX HIN200 domain (306-RRAKKIPKINILHKQTSGYIVYGLFMLHTKIVNRK-340, score 4.4) (Fig. S2C). We did not pursue this putative bipartite motif for several reasons. First, the low score assigned by NLSapper made this putative NLS motif an unlikely candidate. Second, the HIN200 domain is known to require its basic residues to form electrostatic interactions with the negatively charged sugar-phosphate DNA backbone (8). Therefore, a deletion mutant could impact its ability to bind DNA. Third, when aligning the IFIX amino acid sequence with those of the other human PYHIN proteins, we noted that the putative IFIX NLS motif-2 and -3 shared significant homology with IFI16 and MNDA (Fig. 5C). These two motifs shared no homology with AIM2, which is strictly localized to the cytoplasm, while there was alignment in and adjacent to the NLS motif-1 (Fig. 5C). In agreement with a likely conserved function for these motifs in regulating the localization of PYHIN proteins, we previously identified the sequence 138-KRKK-141 as motif-2 within the bipartite IFI16 NLS (31). Of note, this is also the region Ding et al. described as being the putative IFIX NLS when IFIX was initially discovered (23), although this motif was not experimentally tested at that time. Therefore, we focused on further characterizing the partial cytoplasmic phenotypes of ΔNLS1, ΔNLS2, and ΔNLS3.

### K138 acetylation within the NLS motif-2 induces partial IFIX cytoplasmic localization.

**FIG 6:**
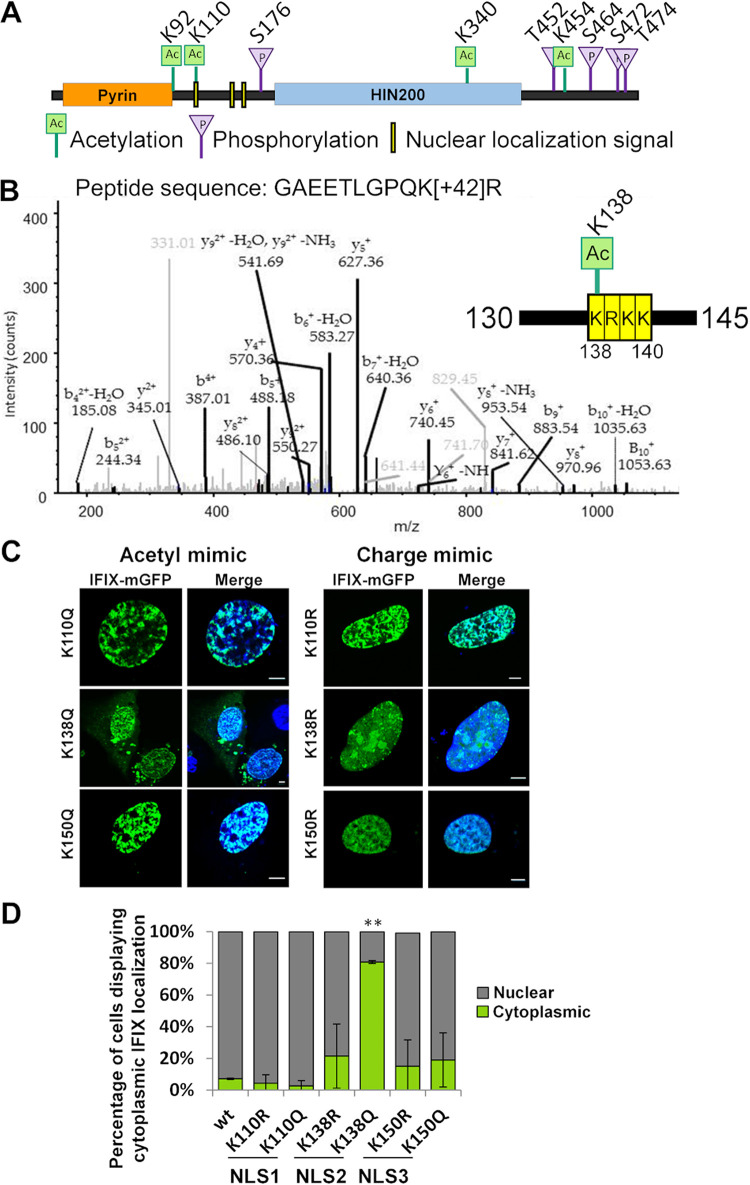
IFIX NLS acetylation at K138 modulates its subcellular localization. (A) Schematic of the IFIX protein displaying PTMs that were identified through DDA MS analysis. Ac in green square, acetylation; P in purple triangle, phosphorylation; NLS motifs are indicated by yellow boxes. (B) Representative spectrum of the identified acetylated K138 peptide. Inset schematic shows localization of K138 acetylation within NLS motif-2. (C) Comparison of IFIX localization upon mutation of residues within the NLS that are acetylated. U2OS cells were transfected with the indicated constructs and then imaged. Acetyl mimics (left) are compared with charge mimics (right). Scale bars, 5 μm. (D) Quantification of acetyl mimic cytoplasmic localization (percentage of cells). Values are means ± SD from two biological replicates; *n *≥ 25; **, *P* ≤ 0.005.

For the related PYHIN protein IFI16, we previously found that acetylation on lysine 99 in motif-1 and lysine 128 in motif-2 induce the localization of IFI16 to the cytoplasm (31). Since there is considerable conservation of these basic residue clusters within the predicted NLSs between IFI16 and IFIX (Fig. 5C), we reasoned that acetylation might also contribute to cytoplasmic localization of IFIX. However, although different types of posttranslational modifications (PTMs) have been identified for IFI16 and other nuclear and cytoplasmic DNA sensors (35), no PTMs have been reported for IFIX in any biological context. Therefore, to determine whether IFIX is posttranslationally modified, we enriched IFIX-mGFP by immunoaffinity purification (IP) and performed mass spectrometry analyses. We identified several acetylation and phosphorylation sites that were distributed in particular in the linker region between the pyrin and the HIN domain and at the C terminus of IFIX (Fig. 6A). Among the identified PTMs, the acetylation on lysine 110 resides within NLS motif-1 (Fig. 6A). The identification of only one PTM within a predicted NLS motif was not surprising considering that the ratio of cytoplasmic-to-nuclear IFIX levels is very low, even during DNA sensing (as shown in Fig. 4A). Hence, a PTM that may promote cytoplasmic IFIX localization might not be abundant enough to be detected via data-dependent acquisition (DDA) by mass spectrometry. To address this, we utilized a targeted MS approach termed selected reaction monitoring (SRM) and designed this assay to specifically search for the presence of acetylated K138 within NLS motif-2. Our alignment of PYHIN protein sequences showed a high level of conservation for NLS motif-2 between IFI16, IFIX, and MNDA (Fig. 6C). This region contains the lysine residue that we previously identified to regulate IFI16 localization (K128 in IFI16 corresponding to K138 in IFIX) (31). Given that we now determined that the NLS motif-2 also impacts IFIX localization (Fig. 5B), we asked whether this residue can also become acetylated in IFIX. To promote IFIX NLS acetylation, we induced expression of IFIX-GFP in 293 cells, transfected with VACV70, and then isolated IFIX via IP. Indeed, the SRM analyses provided evidence of IFIX acetylation at K138 (Fig. 6B).

To investigate the effects of lysine acetylation within each putative IFIX NLS, we next turned to mutational analyses. The lysine (K) residues were replaced with either glutamine (Q) residues as acetyl mimics or with arginine (R) residues as mimics of the unmodified positively charged lysines. Constructs were generated for K110 and K138, the two sites identified to contain acetylations in motif-1 and motif-2, respectively. Additionally, we also constructed mutants for the K150 residue within motif-3, given the high similarity of this motif between IFIX and IFI16 (KRSK versus KGSK, respectively) (Fig. 5C). We transfected these mutants into U2OS cells and monitored IFIX localization by confocal microscopy. Our results showed that while the acetyl mimics K110Q and K150Q within motif-1 and -3 had no significant impact on IFIX localization, the K138Q mutation increased the ratio of cytoplasmic-to-nuclear IFIX distribution (Fig. 6C). Quantification of biological triplicates, *n* ≥ 50 cells each, revealed that K138Q displayed cytoplasmic levels of IFIX, similar to the full deletion of motif-2 (Fig. 6D compared to 5B). Additionally, none of the charge mimics, including K138R, showed significant disruption of IFIX localization (Fig. 6C and D). Altogether, these data suggest that acetylation of K138 impacts IFIX localization, pointing to a conserved regulatory hub of PYHIN protein localization via the acetylation of this NLS motif.

## DISCUSSION

As mammalian cells face a myriad of invading pathogens every day, it is critical that they effectively recognize and respond to infections. To accomplish this feat, cells employ an array of PRRs that prime the innate immune system to stem the spread of infection. One challenge for PRRs is that different pathogens replicate within distinct compartments in the cell. As such, cells express a variety of PRRs that are localized to the nucleus, cytoplasm, endosomes, plasma membrane, etc., to capture a variety of PAMPs. However, accumulating evidence has uncovered that the localization of PRRs is not static. For example, the prominent cytoplasmic DNA sensor cyclic GMP-AMP synthase (cGAS) has also been shown to act within the nucleus and to even synergize with another nuclear DNA sensor, IFI16 (36–38). Furthermore, IFI16 was initially discovered as a cytoplasmic DNA sensor (10), and our lab discovered that its predominantly nuclear localization is regulated by acetylation within a bipartite NLS (31). Thus, to better understand the function of a DNA sensor, we must investigate both its localization when the cell encounters foreign DNA in different compartments and the immune response that is induced upon DNA sensing.

Here, we performed the first analysis of host cell proteome changes that require a nuclear DNA sensor. Following two forms of innate immune system stimulation, HSV-1 infection and VACV 70-mer transfection, we isolated nuclear and cytoplasmic fractions in a CRISPR-mediated IFIX knockout background compared to cells expressing IFIX. Given the observed induction of *ifn-β* expression by IFIX upon both HSV-1 infection and VACV 70-mer transfection (Fig. 1B) (11), it is possible that proteins involved in immune response would be modulated upon IFIX knockout. Such changes may be subtle given the known ability of HSV-1 to suppress immune signaling, which is, in part, mediated by broad shutoff of host transcription and translation by viral proteins (39–41). As both WT and IFIX-KO cells would encounter protein synthesis shutoff induced by HSV-1 infection, this experimental design allowed us to determine proteome changes stimulated by IFIX regardless of viral immune evasion.

Indeed, our results pointed to a subset of proteins whose abundances were lower in the IFIX-KO cells during HSV-1 infection, which included proteins involved in immune signaling and vesicle-mediated transport. Among these was CACTIN, LRRFIP2 (Leucine-rich repeat flightless-interacting protein 2), and ERC1 (ELKS/Rab6-interacting/CAST family member 1), proteins involved in Toll-like receptor (TLR) and/or NF-kappa-B signaling, as well as TTLL12 (Tubulin-tyrosine ligase-like protein 12), a protein linked to RIG-I signaling. Additionally, the abundances of the nuclear DNA sensors IFI16 and hnRNPA2B1 became slightly elevated in the IFIX-KO cells at 6 hpi (Fig. 3B). It remains to be determined whether this upregulation represents a compensatory mechanism by the cells for the lack of IFIX to maintain nuclear DNA sensing and induction of antiviral responses. The synergies between nuclear DNA sensors have not yet been elucidated in detail, and future studies can help to better define the unique, redundant, and cooperative functions of these proteins. Interestingly, the abundance of OASL, a protein linked to cytoplasmic RNA sensing, was elevated in IFIX-KO cells upon VACV70 transfection (Fig. 4B). We previously demonstrated that OASL negatively regulates activity of the cytoplasmic DNA sensor cGAS during HSV-1 infection (29). The possible cross talk between the DNA and RNA sensing pathways has only recently started to come to light, and whether multiple connections exist between proteins functioning in these pathways remains unknown. Compensatory mechanisms could exist here as well. Conversely, when activated by the presence of VACV70 in the cytoplasm, nuclear IFIX may function to transcriptionally suppress the levels of OASL to prevent cGAS inhibition. Alternatively, IFIX-mediated immune signaling could function to suppress OASL expression and RNA sensing.

Given that IFIX, a predominantly nuclear protein, both binds VACV70 in the cytoplasm and influences the abundance of a multitude of cytoplasmic proteins, we next examined the IFIX domains and properties that regulate its subcellular localization. Bioinformatic predictions, followed by experimental investigations, pointed to the contribution of multiple NLS motifs to nuclear localization of IFIX. However, neither motif proved sufficient for this localization task. Furthermore, deletions of all three putative NLS motifs did not fully abolish IFIX levels in the nucleus (Fig. 5B), and there are other clusters of basic residues within IFIX that could function as nonclassical NLSs. An important consideration here is that it remains to be seen whether these deletion mutations affect the stability of the protein in either the nucleus or the cytoplasm. Although such differences in stability could bias the analysis, quantification of the fluorescent signals indicated similar expression levels for the WT and NLS deletion mutants. It is also possible that other localization sequences support interactions with the nuclear import machinery or that IFIX has an NLS that spans multiple protein domains. Examples of the latter have been previously reported for other proteins (42). In support of this, NLSmapper identified a putative NLS within the IFIX HIN200 domain (see Fig. S2 in the supplemental material), but the significance of this sequence has not yet been tested. The observed redundancy in NLS activity may reflect the cellular need for IFIX protein in the nucleus, although IFIX homeostatic functions remain unknown. Taken together, these data suggest that IFIX is regulated by a multipartite NLS.

Protein acetylation is a posttranslational modification that can impact protein function and localization via different mechanisms (43). We previously determined that the related PYHIN protein, IFI16, is acetylated within a bipartite NLS, a modification that results in the cytoplasmic localization of IFI16. In this study, we identified lysine acetylations within IFIX NLS motifs at K110 and K138, which share amino acid sequence homology with IFI16. Our hypothesis that acetylation of the NLS can also modulate IFIX localization was supported by the cytoplasmic enrichment (80% of cells) of K138Q acetyl mimic IFIX. Interestingly, although NLS2 and NLS3 appeared to be equally important for promoting nuclear IFIX localization, the K150Q acetyl mimic had no effect on IFIX localization. Beyond acetylation, phosphorylation has also been recognized to regulate the nuclear import of proteins (42). Phosphorylation of proteins within NLS sequences can decrease the binding affinity of a protein for importin alpha, thereby decreasing protein abundance in the nucleus (44). Although we did not identify any phosphorylation of IFIX in the immediate vicinity of the NLS motifs, the NLS motif-3 (KRSK) does contain a serine at the third position, which could be phosphorylated to regulate IFIX localization. Additionally, we found that a serine residue proximal to NLS motif-3, S176, was phosphorylated, and future investigations of this modification are necessary to determine its role in IFIX localization. Future studies will also show whether this region could contain additional posttranslational modifications, including phosphorylation, acetylation, SUMOylation, and ubiquitination, which could be context dependent (e.g., different stimuli and cell types). Another outstanding question is what mechanism drives the acetylation of the IFIX NLS: what enzymes regulate this site, and what signal initiates the addition of this PTM? Our findings uncover NLS acetylation within an NLS motif as a conserved regulatory hub for modulating the subcellular localization of nuclear DNA sensors from the PYHIN family, and future studies can help to determine whether this represents a broader mechanism for shuttling immune factors between subcellular compartments.

## MATERIALS AND METHODS

### Reagents.

The antibodies used for immunofluorescence, immunoblotting, and immunoaffinity purifications were α-pH2A.X (JBW301; EMD Millipore), α-tubulin (T6199; Sigma-Aldrich), α-H3 (ab1791; Abcam), and a rabbit polyclonal α-GFP antibody generated in-house. Alexa Fluor secondary antibodies (Thermo Fisher Scientific) were used for both Western blotting (680 goat α-mouse and 800 goat α-rabbit) and immunofluorescence (568 goat α-mouse). Labeled and unlabeled vaccinia virus 70-mer (VACV70) and ISD oligonucleotides were both synthesized by Integrated DNA Technologies and annealed as previously described (11). All transfections of DNA constructs were performed using Lipofectamine 2000 (Life Technologies) according to the manufacturer’s instructions.

### Cell culture and viruses.

Primary human foreskin fibroblasts (HFFs), U2OS, and HEK293 Flp-In T-Rex cells were cultured in Dulbecco’s modified Eagle’s medium (DMEM) (Sigma-Aldrich) supplemented with 10% (vol/vol) fetal bovine serum (Atlantic Biologicals) and 1% (vol/vol) penicillin-streptomycin (Gibco) at 37°C in 5% CO_2_. HEK293 FLp-In T-Rex cells were a gift from Loren W. Runnels of University of Medicine and Dentistry of New Jersey/Robert Wood Johnson Medical School (New Brunswick, NJ, USA). HFFs were a gift from Hillary Coller of University of California–Los Angeles (Los Angeles, CA, USA). For all experiments using inducible 293 cell lines, cells were treated with 1 μg/ml tetracycline for 16 to 24 h to induce expression of IFIX-GFP prior to their use in experiments.

Viruses used were strain 17+ wild-type HSV-1 (WT HSV-1), a gift from Saul Silverstein (Columbia University, New York, NY), and HSV-1::*mrfp-vp26*, provided by Lynn Enquist (Princeton University, Princeton, NJ). Viruses were grown in U2OS cells and collected when 100% of cells exhibited cytopathic effect. To harvest virus, both culture supernatant and cell-associated virus were collected, and the cell-associated virus samples were sonicated and centrifuged to pellet cell debris. Supernatants were then combined over a 10% Ficoll cushion and purified via ultracentrifugation. Virus pellets were resuspended in MNT buffer (200 mM morpholineethanesulfonic acid, 30 mM Tris-HCl, 100 mM NaCl, pH 7.4) and titers determined by plaque assay on U2OS cells. For infections, virus (or no virus for mock infection) was diluted in DMEM containing 2% (vol/vol) fetal bovine serum (FBS) and incubated on cells at the indicated multiplicity of infection (MOI) for 1 h at 37°C, 5% CO_2_ with gentle rocking every 15 min to allow for virus adsorption. Cells were then washed once with phosphate-buffered saline (PBS), overlaid with the complete medium described above, and incubated for the indicated lengths of time.

### Construction of stable cell lines.

Primary human fibroblasts stably expressing mGFP or IFIX-mGFP were generated previously in our laboratory via retrovirus transduction with pLXSN plasmids (24). The inducible IFIX-GFP 293 cells were generated previously in our laboratory by cotransfection with pcDNA5/FTR/TO plasmids (11). IFIX-KO and control HFF cell lines were produced using TrueCut Cas9 protein V2 (Thermo Fisher Scientific) and CRISPRMAX Cas9 transfection reagent (Thermo Fisher Scientific) with TrueGuide synthetic guide RNA (negative control, nontargeting 1; PYHIN1, CRISPR728173_SGM; Thermo Fisher Scientific) according to the manufacturer’s instructions.

### Plasmids and mutagenesis.

Site-directed mutagenesis was performed with a QuikChange kit (Agilent) by using the pEGFP-N1 plasmid containing IFIX as a backbone followed by DpnI digestion to generate IFIX acetyl mimics as well as NLS deletion mutants.

### Immunofluorescence staining and microscopy.

Cells were seeded in a 35-mm glass-bottom culture dish (MatTek) and were subjected to the indicated infections, transfections, and UV light treatment described above and fixed at the indicated time points. To induce DNA damage cells were left untreated or treated with UV at the indicated energy (Stratalinker; Agilent Technologies) and then fixed 24 h after treatment. Cells were fixed with 4% paraformaldehyde in PBS for 15 min and permeabilized with 0.1% Triton X-100 in PBS for 15 min. Three washing steps with PBS containing 0.2% (vol/vol) Tween 20 (PBS-T) were performed after fixation and again after permeabilization. For indirect fluorescence, the cells were blocked with 3% (wt/vol) bovine serum albumin (BSA) and 3% (vol/vol) human serum in PBS-T for 1 h at room temperature. The cells were then stained sequentially for 1 h at room temperature with the indicated primary and secondary antibodies in blocking solution. After incubation with secondary antibody, cells were stained with 1 μg/ml 4′,6-diamidino-2-phenylindole (DAPI) in PBS-T for 10 min. Cells were washed three times with PBS-T after each incubation, stored, and imaged in Dulbecco’s PBS.

Cells were visualized with either a Nikon A1 confocal microscope or a Nikon Ti-E equipped with a Yokogawa spinning disc (CSU-21) and digital camera (Hamamatsu ORCA-Flash TuCam). For live-cell imaging experiments, cells were maintained at 37°C and 5% CO_2_ using an environmental control chamber. For quantification of IFIX NLS and acetyl mimic mutants, 3 biological replicates were performed (*n* ≥ 50 total cells) and significance was determined using Student's *t* test relative to the WT IFIX construct. For all experiments, cells were imaged with either 60× or 100× oil immersion objectives. Microscopy images shown in this work were processed using ImageJ (National Institutes of Health). All scale bars correspond to 5 μm in length.

### Immunoblotting.

Cells were collected for Western blot analysis in Laemmli buffer with 100 mM dithiothreitol (DTT) and boiled at 95°C for 5 min. All samples were resolved by 10% SDS-PAGE. Proteins were transferred to polyvinylidene difluoride (PVDF) membranes and blocked in 5% milk in Tris-buffered saline with (TBS) at room temperature for 1 h. Membranes were incubated with primary antibody in blocking solution with 0.2% (vol/vol) Tween 20 for 1 h at room temperature. Fluorescently conjugated secondary antibody incubations were performed at room temperature for 45 min in blocking solution with 0.2% (vol/vol) Tween 20 and 0.05% (wt/vol) SDS. Membranes were washed twice in TBS with 0.2% (vol/vol) Tween 20 and once with TBS before visualizing using an Odyssey CLx imager (LI-COR Biosciences).

### cDNA generation and quantitative PCR.

RNA extraction was performed using an RNeasy kit (Qiagen) according to the manufacturer’s instructions. DNA was digested using DNase I, and cDNA was generated via reverse transcription-PCR (RT-PCR) using the Superscript IV first-strand cDNA kit (Thermo Fisher Scientific) according to the manufacturer’s instructions. For quantitative PCR, amplification was performed using Power SYBR green PCR master mix (Life Technologies, Inc.) and gene-specific primers with a Viia7 thermocycler (Thermo Fisher Scientific). Relative quantification was assessed by the ΔΔ*C_T_* method using either *β-acting* or *gapdh* as the reference gene.

### Bioinformatics.

NLSmapper (32) was used to identify putative NLS motifs within the IFIX sequence. PYHIN amino acid sequence alignments were performed using Clustal Omega and visualized with Jalview (45).

### Immunoaffinity purification for the identification of IFIX PTMs.

IFIX PTMs were determined following enrichment of IFIX via immunoaffinity purification (IP) followed by nLC-tandem MS (nLC-MS/MS) analysis. IFIX-GFP 293 cells were induced with tetracycline (1 μg/ml) for 18 h and then washed and scraped in PBS, resuspended in freezing buffer (20 mM Na-HEPES, 1.2% polyvinylpyrrolidone [wt/vol], pH 7.4), and flash frozen as cell pellets in liquid nitrogen. For targeted mass spectrometry to identify lysine 138 acetylation, cells were transfected with VACV70 (1 μg/ml) for 3 h prior to harvesting cells. Frozen pellets were then ground using a Retch MM301 mixer mill (Retch, Newtown, PA) for 1.5 min at 30.0 Hz for 8 rounds with recooling in liquid nitrogen between rounds, as described previously (46). Each replicate was resuspended in 5 to 7 ml of optimized lysis buffer (20 mM K-HEPES, pH 7.4, 0.11 M potassium acetate [KOAc], 2 mM MgCl_2_, 0.1% Tween 20 [vol/vol], 1 μMZnCl_2_, 1 μM CaCl_2_, 0.6% Triton X-100, 200 mM NaCl, 1:100 phosphatase inhibitors [Sigma], 1:100 protease inhibitor cocktail [Sigma], and 100 U/ml Benzonase [Pierce]) for DNA and RNA digestion. A PT 10–35 GT Polytron (Kinematica, Bohemia, NY) was used for 20 s at 20,000 rpm to homogenize the samples, followed by centrifugation at 8,000 × *g* for 10 min at 4°C. Six milligrams of M-270 epoxy magnetic beads (Life Technologies) that were conjugated with our in-house-generated polyclonal GFP antibody as in reference 47 were used for each IP. Bead incubations were for 1 h at 4°C, after which the beads were washed 5 times with lysis buffer and twice with PBS. The coisolated proteins were eluted in 1× lithium dodecyl sulfate sample buffer (Life Technologies) by incubating the beads in a thermomixer at 70°C with agitation (1,500 rpm) for 10 min, followed by an additional 10-min agitation on a TOMY shaker at room temperature.

### MS sample preparation for PTM identification.

Isolated IFIX samples were reduced and alkylated with 0.05 M final concentrations of each Tris(2-carboxyethyl)phosphine hydrochloride (TCEP) (Pierce) and chloroacetamide (CAM) by heating at 70°C for 20 min. SDS-PAGE (4 to 12% Bis-Tris NuPAGE gel) was used to partially resolve proteins and was then stained with SimplyBlue Coomassie safe stain (Life Technologies) overnight and destained by Milli-Q water washes until the background became clear. Proteins were processed through in-gel protein digestion by cutting gel lanes into 1-mm-thick cubes and processed using an in-gel digestion protocol, as described previously (18). Samples were digested with 12.5 ng/μl trypsin (Promega, Madison, WI) overnight at 37°C. Peptides were extracted by incubating the gel pieces in 0.5% formic acid for 1 h at room temperature with agitation, followed by 1 h at room temperature without agitation. A second extraction was performed by incubating the gel pieces in 0.5% formic acid–50% acetonitrile for 2 h at room temperature and pooled with the first extraction. Vacuum centrifugation was utilized to remove acetonitrile from the extracted peptides. Peptides were then acidified to 1% trifluoroacetic acid (TFA) and desalted using StageTips assembled from low-retention plastic tips (Eppendorf) and SDB-RPS Empore Discs (Sigma-Aldrich). StageTip membranes were washed with 0.2% TFA and eluted into autosampler vials with 5% ammonium hydroxide–80% acetonitrile. Samples were vacuum centrifuged to 1 μl and resuspended in 8 μl of 1% formic acid–4% acetonitrile.

### MS sample preparation for nuclear-cytoplasmic proteomes.

Samples used for proteome analysis were collected at the indicated times postinfection and -transfection. Briefly, cells were washed and scraped in PBS with 1:100 Halt protease and phosphatase inhibitor cocktail (P/PhIC; Thermo Fisher Scientific) and then flash frozen as cell pellets in liquid nitrogen. Cells were thawed and lysed in lysis buffer 1 (50 mM HEPES-KOH, 140 mM NaCl, 1 mM EDTA, 10% glycerol, 0.5% NP-40, 0.25% Triton X-100, and 1:100 P/PhIC). Nuclei were pelleted by centrifugation at 1,350 × *g* for 10 min at 4°C. Nuclei were then washed with lysis buffer 2 (10 mM Tris-HCl, pH 8.0, 200 mM NaCl, 1 mM EDTA, 0.5 mM EGTA, and 1:100 P/PhIC), spun down again, and lysed in lysis buffer 3 (10 mM Tris-HCl, pH 8.0, 100 mM NaCl, 1 mM EDTA, 0.5 mM EGTA, 0.1% Na-deoxycholate, 0.5% N-lauroylsarcosine, 1:100 P/PhIC). Both cytoplasmic and nuclear lysates were brought to 5% SDS and then boiled at 95°C three times for 5 min with sonication in between. Protein concentrations were measured with the Pierce bicinchoninic acid protein assay kit (Thermo Fisher Scientific) according to manufacturer’s instructions. Ten micrograms of protein was then reduced and alkylated with 50 mM CAM and 25 mM TCEP before adding aqueous phosphoric acid to a final concentration of ∼1.2%. Digestion was then performed with 0.75 μg trypsin and suspension trapping columns (S-Trap; Protifi) for 1 h by following the manufacturer’s instructions. Peptides were then resuspended in 5 μl 1% FA, 1% acetonitrile.

### MS acquisition and analysis.

For the identification of IFIX PTMs, 4 μl of each sample was used for analysis using nLC-MS/MS using a Dionex Ultimate 3000 nanoRSLC (Dionex Corp., Sunnyvale, CA) coupled online to an EASY-Spray source and an LTQ-Orbitrap Velos (Thermo Fisher Scientific, Waltham, MA). A 50-cm-by-75-μm-inner-diameter PepMap RSLC C_18_ 2-μm (Thermo Fisher Scientific) EASY-Spray column was used for the reverse-phase chromatography separation of peptides at a flow rate of 250 nl/min over a 90-min gradient of acetonitrile consisting of 4% to 40% B (mobile phase A, 0.1% formic acid in water; mobile phase B, 0.1% formic acid in 97% acetonitrile). Peptide precursors were subjected to collision-induced dissociation (CID) MS/MS fragmentation in the ion trap for the 15 most abundant precursor ions (data-dependent acquisition).

For SRM to detect IFIX lysine 138 acetylation, an IFIX spectral library was first generated in Skyline (v.3.1.1.9007), and Skyline was used to calculate the theoretical mass of the tryptic peptide with K138 acetylation. Peptides were separated by nLC and injected onto an LTQ-Orbitrap Velos as described above but with a gradient of 1 to 40% B. Full scans had an isolation width of 2, with normalized collision energy of 35 and maximum ion time of 150 ms, and were attained in the dual-pressure linear ion trap from the MS/MS mass inclusion list (generated from Skyline). One MS spectrum from the inclusion list of the full scan was collected in the Orbitrap after each MS/MS cycle. Resolution was set to 7,500 with an *m/z* range of 350 to 1,700.

For analysis of nuclear and cytoplasmic proteomes, peptides were analyzed by nano-liquid chromatography coupled to tandem mass spectrometry with a Q Exactive HF hybrid quadrupole-Orbitrap instrument (Thermo Scientific) using data-dependent (DDA) mode. Peptides were injected in 2-μl volume and separated with a 3% solvent B to 35% solvent B gradient (solvent A, 0.1% FA; solvent B, 0.1% FA, 97% ACN) over 150 min at a flow rate of 250 nl/min on an EASYSpray C_18_ column (75 μm by 50 μm) heated to 50°C. The full scan range was set to 350 to 1,800 *m/z* at 120,000 resolution and recorded in profile. The top 20 most intense precursors were subjected to HCD fragmentation-normalized collision energy (NCE) of 28 for MS2 analysis at 15,000 resolution with automatic gain control (AGC) target set to 1e5, 42-ms maximum injection time, and an isolation window of 1.2 *m/z*.

### IFIX protein and PTM identification.

Raw data from MS analyses of immunoisolations were extracted and searched against UniProt Swiss-Prot sequence databases that included common contaminants (downloaded August 2013) in Proteome Discoverer (v. 1.4.0.288; Thermo Fisher Scientific) using the SEQUEST HT node (v1.3; Thermo Fisher Scientific). The following criteria were used as search parameters: full trypsin specificity, maximum 2 missed cleavage sites, and precursor and fragment ion mass tolerance of 10 ppm and 0.5 Da, respectively. The following dynamic modifications were searched: acetylation, oxidation, and phosphorylation. The static modification carbamidomethylation was allowed. Percolator in Proteome Discoverer was used to calculate peptide spectral match probabilities against a decoy database. IFIX protein and PTM identification was performed in Scaffold (v. 4.4.8; Proteome Software, Inc.) using X!Tandem (48) and ProteinProphet (49) algorithms. PeptideProphet (50) was used for probabilistic validation of peptide identifications, and the corresponding protein probabilities were derived using ProteinProphet in Scaffold. Protein- and peptide-level probability filters were set to 1% false discovery rate with at least 2 unique peptides in at least one biological replicate required for protein identification. For SRM data, the Proteome Discoverer search was performed as described as above except using a wide ppm percursor tolerance. Validation of the acetylation was done manually by inspecting individual spectra and matching any background to precursor ions in the full scan.

For nuclear and cytoplasmic proteomes, tandem MS spectra collected from DDA mode were analyzed by Proteome Discoverer v2.4. The Spectrum Files RC node was used to perform offline mass recalibration, and the Minora Feature Detector node was used for label-free MS1 quantitation. MS/MS spectra were searched using a precursor and fragment mass tolerance of 5 ppm and 0.02 Da using the SEQUEST HT algorithm against a UniProt human database containing herpesvirus sequences and common contaminants (downloaded January 2021). PTMs, including static carbamidomethylation of cysteine, and dynamic oxidation of methionine, loss of methionine plus acetylation of the protein N terminus, acetylation of the protein N terminus, and glutamate to pyroglutamate conversion, were specified. The Percolator node was then used to perform peptide spectrum match (PSM) validation, and the IMP-ptmRS node was used for assigning PTM site probabilities. PSMs were assembled into peptide and protein identifications in the consensus report with a false discovery rate of less than 1% at the peptide and protein level. MS1-based quantitative values were normalized between samples based on the total peptide signal. Normalized protein abundances were calculated from the sum of valid normalized peptide abundances, quantified in at least 2 out 3 replicates and with missing values imputed using replicate-based resampling. Proteins with at least 2 quantified peptides in six samples (*N* = 3 IFIX-KO and *n* = 3 controls) from at least one condition (mock, 3 hpi, 6 hpi, or VACV) were retained and exported to Excel for further processing.

### MS data processing and bioinformatics.

Protein abundances were imported into Perseus software (v 1.6.14.0) (51). Abundances were log transformed, and missing values were imputed using QRILC (default settings) from the ImputeLCMD R package (52). Differential abundance was assessed using the Volcano plot function (default settings), except with a more stringent FDR threshold of 2.5%. Hierarchical clustering and heatmap construction were performed with Clustvis (53), which performed row centering and univariate scaling (for abundances), followed by row clustering using distance measures of either correlation (abundances) or Euclidean (ratios) and Ward.D2 distance linkage. Gene ontology enrichment was performed using either clusterProfiler (54) or STRING (55). Functional protein networks were assembled using the STRING app (default settings) in Cytoscape (ver. 3.8.2) (56). InstantClue (57) and GraphPad Prism (ver 9) were used for data visualization and statistical analysis.

### Criteria for manual inspection of MS/MS spectra.

MS/MS spectra that were assigned to acetyllysine-containing peptides were manually inspected. (i) The presence of a continuous b or y-ion series of at least five residues was inspected. (ii) The majority of intense fragment ions were assigned (within a mass accuracy of ±0.4 Da) to singly or doubly charged b or y ions or b or y ions resulting from a neutral loss of water or ammonia. (iii) A site-determining fragment ion was assigned. (iv) The sequence assignment reflected a missed trypsin cleavage C-terminal to the acetyl lysine.

### Data availability.

The data-dependent mass spectrometry proteomics data have been deposited in the ProteomeXchange Consortium via the PRIDE (58) partner repository with the data set identifier PXD025101. The SRM mass spectrometry data have been deposited at PASSEL (59) with the identifier PASS01677 and can be accessed at http://www.peptideatlas.org/PASS/PASS01677.
