# Supplementary figures and images for: The DNA Sensor IFIX Drives Proteome Alterations To Mobilize Nuclear and Cytoplasmic Antiviral Responses, with Its Acetylation Acting as a Localization Toggle
Source: mSystems. 2021 Jun 22;6(3):e00397-21. doi: 10.1128/mSystems.00397-21 (PMC8269231; doi:10.1128/mSystems.00397-21)

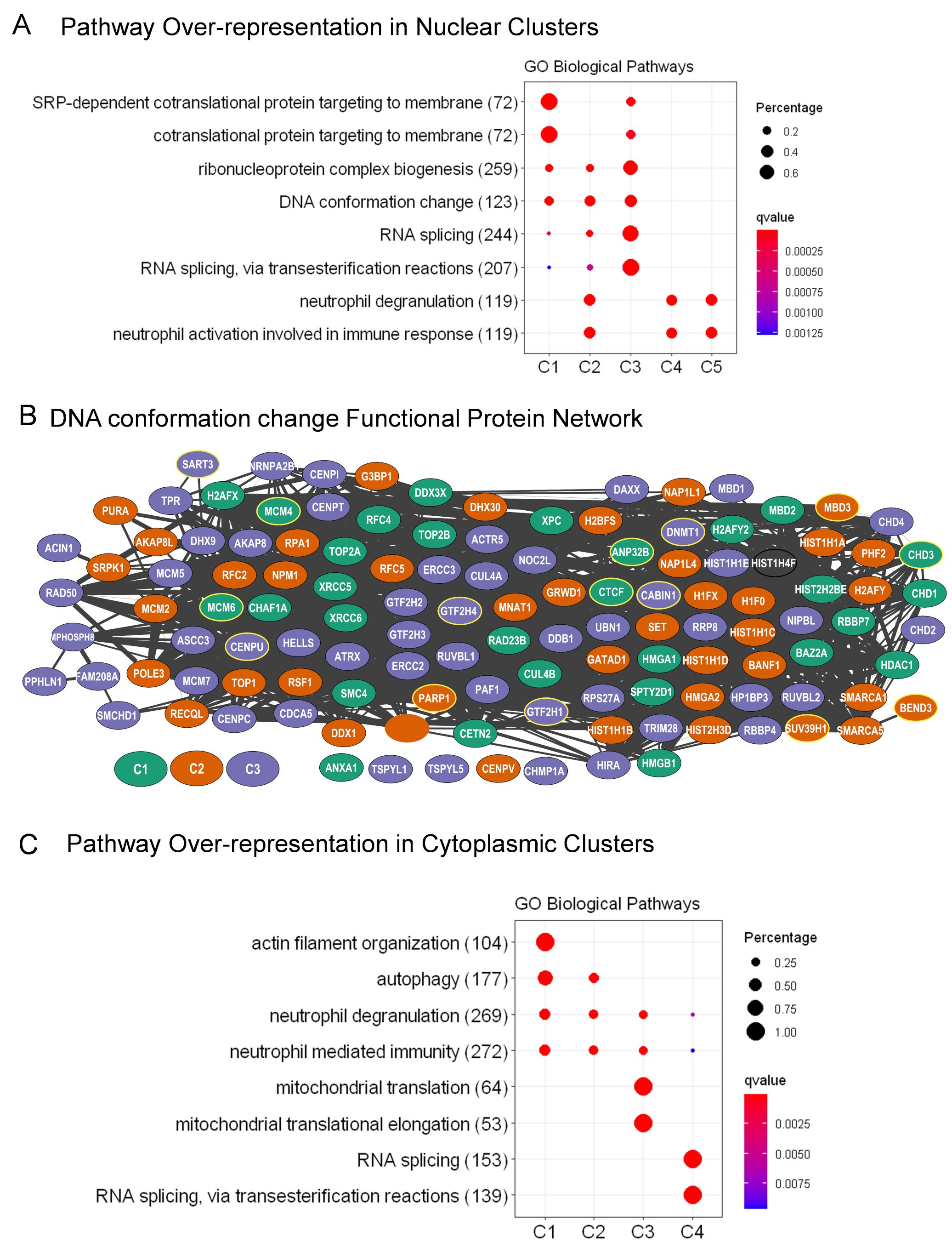

Supplement: FIG S1 [file msystems.00397-21-sf001.tif]

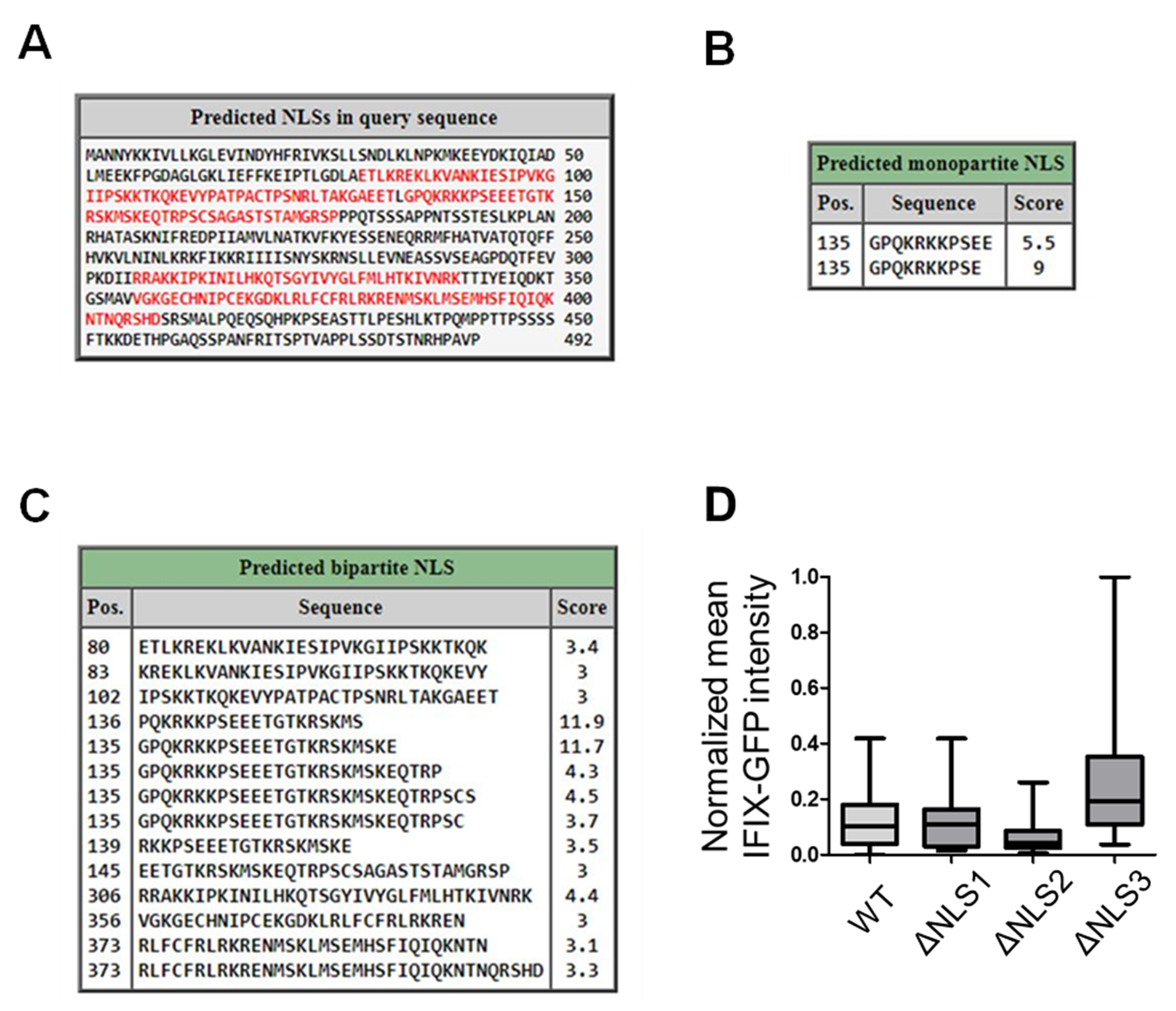

Supplement: FIG S2 [file msystems.00397-21-sf002.tif]
